# Effect on Glycemic Control of an Early Intensive Dietary Structured Education Program for Newly Diagnosed Children with Type 1 Diabetes in Jordan

**DOI:** 10.1155/2023/7258136

**Published:** 2023-04-28

**Authors:** Abeer Alassaf, Lobna Gharaibeh, Sarah Ibrahim, Shatha Alkhalaileh, Rasha Odeh

**Affiliations:** ^1^Department of Pediatrics, The University of Jordan, Amman, Jordan; ^2^Pharmacological and Diagnostic Research Center, Faculty of Pharmacy, Al-Ahliyya Amman University, Amman, Jordan

## Abstract

**Methods:**

This is a retrospective medical chart review study at Jordan University Hospital. The glycemic control of children who were diagnosed with T1D and included in the SEP between June 2017 and December 2019, was compared with those who were exposed to the conventional diabetes education, between January 2014 and December 2016. Various factors were assessed for the possible effects on the SEP outcomes.

**Results:**

The average age at diagnosis for the 112 persons with diabetes (PwD) included in the dietary SEP was 8.30 ± 3.87 years. Glycated hemoglobin was lower in children in the SEP group at 6 months (*P* value = 0.001) and 12 months (*P*=0.032) but not at 24 months (*P*=0.290). SEP had better effect on patients older than 5 years. The possible predictors of glycemic control for the SEP group at 12 months included the mother's educational level and the number of hospital admissions due to DKA and hyperglycemia during the first year after diagnosis.

**Conclusion:**

Our dietary SEP was associated with better glycemic control than conventional diabetes education, at 6 and 12 months after diagnosis. It had a positive effect, mainly in PwD patients who are older than 5 years and had higher maternal educational level.

## 1. Introduction

Type 1 diabetes (T1D) is one of the most common chronic diseases in children and adolescents, with significant microvascular and macrovascular complications [1]. An optimal early glycemic control is necessary to decrease the risk of these complications [2]. Diligent efforts are required for daily diabetes care to ensure optimal glycemic control, including testing blood glucose multiple times daily, determining appropriate doses of insulin, and administering it with utmost vigilance, with rapid reactions to low and high readings [3].

Diagnosis with T1D is an overwhelming, stressful experience for patients and their caregivers; it requires psychosocial support and the start of diabetes education as soon as possible. Structured diabetes education is a planned process of providing the persons with diabetes (PwD), and their caregivers with knowledge and skills to enable them to perform the required demanding daily diabetes care and supporting them to feel empowered, while taking into consideration their high levels of stress. This process should be conducted by a devoted multidisciplinary diabetes team of trained health care workers, supervised by quality assurance and continuous adjustment, which includes clinicians, diabetes nurses, dieticians, and social workers [4]. Structured education should be a continuous process, beyond the initial phase after diagnosis, to ensure persistent adherence to the diabetes care plan.

Dieticians have an important role, mainly in dietary advice for healthy meals and advanced carbohydrate counting [5]. It is important to educate PwD and their caregivers on how to determine the amount of carbohydrates in meals in order to calculate the correct doses of insulin, especially for PwD on multiple dose injection (MDI) regimen or pump therapy, as carbohydrates have the major effect on postprandial blood glucose levels [6]. Appropriate doses of insulin are calculated according to the carbohydrate content and blood glucose readings, taking into consideration the factors that affect the blood glucose level such as physical activity [5, 7]. Early dietary diabetes education leads to optimal early glycemic control during the first year after diagnosis, which is known to be tracked in subsequent years and can delay microvascular and macrovascular complications [8].

Various educational programs of advanced carbohydrates counting, that should be started early on after diagnosis, have been developed [9, 10] and are considered an important part of diabetes care in international guidelines [11]. Unfortunately, they are not applied in many countries, for variable reasons, including lack of financial resources and the paucity of dieticians with experience in T1D [12, 13]. Such programs are not widely used in developing countries and even if present, they may not be well-designed or consistent [14]. Consequently, this adversely affects PwD' and their parents' knowledge of the required daily management of diabetes [15].

Personal and clinical factors should be taken into consideration when designing the structured education programs. Barriers to such programs should be identified and overcome by using an individualized approach. The diabetes care team should take into account the socioeconomic characteristics of PwD that may affect their ability to adhere to the required tasks to manage their diabetes [16, 17].

Our center had started a new intensive dietary structured education program (SEP) for newly diagnosed children and adolescents with T1D since June 2017. Our aim was to study the effect of this program on the glycemic control at 6, 12, and 24 months after diagnosis, and to compare the outcomes before and after its implementation. In addition, we examined the effects of certain factors on the outcomes, such as age at diagnosis and socioeconomic characteristics. We hypothesized that our intensive structured dietary program would have a favorable effect on the glycemic control and the frequency of diabetes-related hospital admissions.

## 2. Methods

This was a retrospective chart review study of children and adolescents, who were diagnosed or referred within one month after being diagnosed with T1D to the pediatric endocrine service at Jordan University Hospital, a tertiary academic referral center in the capital Amman, between June 2017 and December 2019. The PwD were given intensive inpatient structured dietary education, followed by close follow-up of a minimum of 3 months after discharge. We compared diabetes outcomes of PwD who were enrolled in the SEP to those who had conventional diabetes care education after diagnosis at our department during the preceding period of SEP (Pre-SEP), between January 2014 and December 2016. The inclusion criteria included PwD who were diagnosed at an age younger than 16 years, followed at least for 2 years by our team. The PwD with celiac disease during the first 2 years after diagnosis were excluded. All PwD were using MDI therapy during the study period. Our study was conducted after the ethical approval from the Jordan University Hospital Institutional Review Board (no.: 2021/311) according to the Declaration of Helsinki, and informed consent was waived as it was a retrospective chart review study. The dietary SEP involved the education of PwD as soon as possible after diagnosis of T1D by the pediatric dietician, who was appointed at our department in May 2017 (Supplementary file 1). The program involved daily individualized sessions for PwD and their caregivers, in addition to practical sessions at the time of meals, mainly breakfast and lunch, with a minimum total daily duration of 2-3 hours for each patient, during their hospital stay, which ranges from 4 to 7 days. The educational sessions included review of basic knowledge of carbohydrates, advanced carbohydrate counting, and the effects of fat and protein in meals, exercise, and intercurrent illnesses on blood glucose levels, and how to adjust insulin doses accordingly. Education involving diabetes self-care management issues other than those involving diet and insulin dosing and dose adjustment, was given by the rest of the diabetes care team. Handouts of instructions and important diabetes care information were given to the PwD during admission. Caregivers were given the contact information of the dietician for mutual communication and close follow-up for blood glucose readings, mainly during the first week after discharge, in addition to answering any question regarding meals and insulin dose calculation. The PwD were then seen at the pediatric endocrine clinic at one, two, four, and eight weeks after discharge. They were seen during the same visit by the dietitian, for around 20 to 30 minutes, to review blood glucose readings, accuracy of insulin dose calculation and review of dose adjustment. Afterwards, they met during the regular clinic visits every 3 months, and then, when needed in between the scheduled clinic visits. Contact with the dietician after the third month became as needed for any question regarding insulin dose calculation and adjustment. Beyond the first year after diagnosis, the PwD were scheduled to see the dietician at least twice per year, to reinforce the knowledge and skills.

Originally, the pediatric endocrine service itself was started at Jordan University Hospital after the joining of the first 2 pediatric endocrinologists (authors) to the pediatric department in February 2012. Prior to that, children and adolescents with T1D were seen by adult endocrinologists, in addition to 2 diabetes nurse educators and a dietician at the internal medicine department. Their experience was mainly with type 2 diabetes in adult patients. Starting February 2012, the pediatric endocrine service started to take care of children and most of adolescents with T1D presenting to the hospital. The 2 attending pediatric endocrinologists covered the main bulk of dietary education, based on international guidelines, including advanced carbohydrate counting, for the newly diagnosed PwD and their caregivers, together with the pediatric residents who were taught this method despite the work load of their clinical duties, as there were no dieticians in our department at that time. The average time spent on diabetes education was 1–2 hours/day during the first 2 days, then supervising carbohydrate counting in all the meals and answering all the questions of PwD and their parents. The adult-diabetes nurse educators met with a number of our PwD and their caregivers once to show them how to use insulin pens and glucometers; they continued to do so after we had started our dietary SEP, but pediatric endocrinologists and pediatric residents had undertaken the main responsibility of hands-on training of how to use those during the hospital admission. The contact numbers of the pediatric endocrinologists were given to parents for questions they may have after discharge till their first clinic visit one week later and afterwards as needed. Clinic scheduling afterwards was similar to the SEP group. Our main aim of designing the dietary structured program was to give the PwD a high-quality diabetes education for advanced carbohydrates counting and calculating and adjusting insulin doses, and to empower them and their parents for diabetes self-care, early on after diagnosis.

We collected demographic, clinical, and socioeconomic data, which included the following: age at diagnosis, gender, glycated hemoglobin (HbA1c), calculated body mass index (BMI) at diagnosis expressed as standard deviation score (SDS), and whether first presentation of T1D was in diabetic ketoacidosis (DKA) or not. In addition to living arrangement, place of residence, the family monthly income documented, and on this basis the group was divided into four categories, namely, less than 400 Jordanian dinars (JD), which is the low-income category, 400–800 JD, 801–1200 JD, and >1200 JD. The type of insurance was divided into the following two categories: Royal Court insurance which covers 100% of the diabetes-related expenses, and the Ministry of Health/University insurance, which covers 85 to 90% of expenses. Insurance covers most of the diabetes-related expenses except for glucose testing strips. The level of education of parents was classified into the following: less than high school, high school, and higher than high school. None of the PwD in our study used continuous glucose monitoring. Other data collected were average daily number of self-monitoring of blood glucose (SMBG) tests during the first year after diagnosis and the person who was responsible for calculating insulin doses during the first year. In addition, the number of clinic visits during the first year after diagnosis and family history of first-degree relatives with type 1 diabetes were also assessed.

The main outcomes measured and compared between the 2 groups of PwD in our study were HbA1c values at 6, 12, and 24 months after diagnosis and diabetes-related hospital admissions (DKA, hyperglycemia, and/or hypoglycemia) during the first 2 years. HbA1c was measured by the Bio-Rad D-10™ Dual Program using an ion-exchange high-performance liquid chromatography. It was expressed in percentage (National Glycohemoglobin Standardization Program (NGSP)) and in mmol/mol (International Federation of Clinical Chemistry (IFCC)). The normal reference range is 4.2–6.2% (22.41–44.27 mmol/mol). The International Society for Pediatric and Adolescent Diabetes recommended glycemic control as reflected by HbA1c of less than 7.5% (58.0 mmol/mol) in children and adolescents in countries with limited resources [18].

### 2.1. Statistical Analysis

Continuous data were presented as the mean ± standard deviation and the categorical data as frequency (%). Associations between dependent categorical variables were evaluated using the Chi-squared analysis. Associations between continuous variables were evaluated using the independent samples *T* test. The univariate and multivariate linear regressions were utilized to assess the possible predictors of HbA1c at 12 months in the SEP group. *P* values less than 0.05 were considered statistically significant. The statistical analysis was performed using the IBM SPSS Statistics for Windows, Version 25 (Armonk, NY: IBM Corp).

## 3. Results

A total of 228 children and adolescents, 129 (56.6%) girls, were included in the study. There were 112 patients (49.1%) diagnosed between June 2017 and December 2019 and included in the SEP group, while 116 children (50.9%) were diagnosed between January 2014 and December 2016, prior to the program (Pre-SEP). The average age of the children was 8.01 ± 3.80 years: 8.30 ± 3.87 years in the SEP group and 7.85 ± 3.75 years in the pre-SEP group. All PwD and their parents spoke the country's official language, in which the program was delivered. No significant difference was seen in dietary habits in both groups, where most PwD have 3 meals and at least one snack over the whole day. The demographic and socioeconomic characteristics in both groups are shown in Table 1, while clinical characteristics are shown in Table 2.

Glycemic control at 6, 12, and 24 months after diagnosis, is shown in Table 3. HbA1c was statistically lower in children in the SEP, compared to those who were not, at 6 months (*P* value = 0.001) and 12 months (*P* value = 0.032). The small sample size may be the reason for some of the statistically insignificant results in Table 3, but not all. Differences in the HbA1c values were significant at both 6 months and 12 months in the analysis of all PwD, where the sample size was large. However, in the two age groups, 5.1–11.9 years and ≥12 years, HbA1c values were statistically significant at 6 months despite the differences in the sample size between the two age groups. Our program appears to be more effective in PwD older than 5 years and in those whose mothers had higher education, with significant reduction in HbA1c at 6 months, but not at 12 or 24 months. On the other hand, there was no significant difference in HbA1c at any time point in patients younger than 5 years.

Possible predictors of glycemic control at 12 months in children in the SEP group were assessed in the univariate and multivariate linear regression model (Table 4). In the univariate model, seven variables were statistically significant, namely, age and HbA1c at diagnosis, HbA1c at 6 months, family monthly income, mother's educational level, place of residence, number of hospital admissions due to DKA, and hyperglycemia during the first year after diagnosis. In the multivariate analysis, results showed that an increase of 1% in HbA1c at 6 months led to an increase of 0.7% in HbA1c at 12 months. Compared to the children with no hospital admissions for DKA or hyperglycemia during the first year, children who had more than 2 admissions had a 1.5% increase in HbA1c at 12 months. Children whose mothers had an education higher than high school compared to those whose mothers had an education less than high school had a decrease of 0.6% in HbA1c. Additionally, children who lived more than 185 kilometers from Amman compared to children who lived in Amman, had an increase of 1% in HbA1c. The percent of PwD who were committed to the scheduled clinic visits in the SEP was 83%. Although the number of clinic visits was comparable between the 2 groups, the major difference was the time spent in the clinic with the dietician in the SEP group.

## 4. Discussion

Our study showed better glycemic control at 6 and 12 months after diagnosis in PwD, who received intensive dietary structured education shortly after diagnosis, compared to those who had received “conventional” education prior to the commencement of the dietary SEP. This is similar to the significant favorable effects on glycemic control due to early structured education reported in previous studies [19].

Although universal guidelines recommend that structured diabetes education programs be started soon after diagnosis [4], several health care settings do not have such programs, and even when present, there could be variable clinical practices for diabetes education [20, 21].

Many factors may play a role in the success or failure of any educational program, including psychosocial factors, program-specific factors, and the health care setting, where the program is conducted. The presence of adequate numeracy skills for PwD and their parents is an important factor in ensuring adherence to advanced carbohydrate counting [22] and subsequent favorable glycemic control [12, 23]. Successful diabetes programs with a focus on carbohydrate counting have been known for many years, as in the Dose Adjustment For Normal Eating (DAFNE) trial, which showed significant improvement in HbA1c at 12 months [9] and in further follow-up studies after several years [24]. It should be noted that the favorable outcomes of an intensive SEP which involves advanced carbohydrate counting may not only be due to accurate calculated insulin doses but also due to positive changes in attitudes of PwD and caregivers, as a result of the program as a whole. Moreover, the other educational programs did not show beneficial effects [25].

Our program did not have significant effect on HbA1c at 24 months after diagnosis. Hawkes et al. [26] reported similar results, where there was a significant positive effect of their program on glycemic control at 6, 12, and 18 months, but this effect was not sustained at 24 months after diagnosis. Essien et al. [27], in a randomized control study, showed encouraging results of their structured education program intervention, on the glycemic control in PwD in Nigeria as an example of low-middle income countries. Other studies in countries with limited resources did not show such favorable outcomes [28]. Similarly, Christie et al. did not find significant long-term effects of their education programs on glycemic control in children with T1D [29]. This may be due to various reasons related to program issues and PwD's adherence. It may be difficult to compare SEPs in various studies, since they vary in type of intervention, duration, and characteristics of patients.

Diabetes education needs to be continuous, with persistent support and supervision, including continuous revision of advanced carbohydrate counting to maintain adequate glycemic control [30, 31]. Our pediatric dietician continued mutual communication with PwD and their parents, addressing their concerns, answering questions, and providing help in diabetes management, which would reinforce their knowledge, increase their confidence, and minimize their fears [32].

Despite the obvious important role of SEP for children with T1D, unfortunately, such programs are lacking among pediatric diabetes care services in developing countries, because of limited resources [33], mainly the scarcity of experienced pediatric diabetes care team members, including dieticians, nurse educators, and social workers.

Our intensive structured dietary program was designed based on international guidelines, adjusted to our population's dietary habits and cultural beliefs, easily delivered by the available one, full-time, pediatric dietician, and under direct supervision of the pediatric endocrinologists. Therefore, it can be applied by other hospitals in Jordan and other developing countries by assigning at least one qualified dietician with experience in T1D, instead of relying on clinicians, who have the work load of clinical duties and variable experience in diabetes education [34].

Our education program did not have significant effect on the incidence of severe hypoglycemia that required admission during the first 2 years after diagnosis, similar to other studies which showed no significant effect on the incidence of hypoglycemia, and no effect on glycemic control as well [35]. However, there are reports that educational programs may play a role in achieving better glycemic control and reducing the incidence of hypoglycemia [25, 36]. Hypoglycemia is an important issue in T1D management, as the fear of its drastic consequences might prevent some families from giving the appropriate doses of insulin, with subsequent suboptimal glycemic control [37]. A systematic review by Schmidt [12] discussed many studies that involved diabetes educational programs, which showed significantly lower or at least no increased incidence of severe hypoglycemia [38]. However, definitions of hypoglycemia and severe hypoglycemia were not uniform in all studies.

There were no significant differences in many demographic and clinical characteristics between the SEP and pre-SEP groups, including gender, age and BMI SDS at diagnosis, and socioeconomic status. This allows better comparison of the effect of the SEP. The presence of unidentified differences between the 2 groups is possible, but they are unlikely to have significant effect on the outcomes, as we investigated the factors mostly known to affect the glycemic control in children with T1D.

The socioeconomic factors have been reported to play a role in attaining favorable glycemic control following diabetes education programs [39]. However, Hawkes et al. did not find positive effect of their education program on the glycemic control in PwD with government insurance, which they considered as a marker for low socioeconomic status [26].

The percentage of PwD whose insulin doses were decided by themselves or their mothers was significantly higher in the SEP group. This may be because mothers are usually the main caregivers, in charge of executing the diabetes care plan and in direct contact with the diabetes clinical team [40, 41], especially when engaged in the SEP. The percentage of PwD with first-degree relatives with T1D at the time of diagnosis was more in the pre-SEP group. Although it might be expected that PwD with first-degree relatives with T1D would have more experience in daily diabetes care, this did not seem a significant factor in achieving better glycemic control compared to acquiring skills through the SEP.

There was no significant difference in the number of clinic visits in both groups, but the time spent in clinic visits was longer in the SEP group, due to the additional time allocated to meet with the dietician and the pediatric endocrinologist. This continued beyond the initial period after diagnosis, similar to a previous study [26].

More frequent SMBG was observed during the first year after diagnosis in the SEP group. This is expected as during this program, frequent testing of blood glucose was required, and discussed during the close, directly supervised follow-up with the diabetes team members [42]. On the other hand, the finding of frequent glucose monitoring might be due to the recall bias as it was self-reported.

There was no significant difference in number of hospital admissions due to hyperglycemia or DKA during the first two years after diagnosis, as had been reported previously [43]. However, one study showed lower incidence of diabetes-related hospital admissions, including DKA, after enrollment in diabetes education programs [9].

The better effect of SEP compared to pre-SEP, in PwD older than 5 years of age and with more educated mothers at 6 months (Table 3), might be due to the possibility of better application of the diabetes care plan by these groups. Hawkes et al. found that SEP benefited children between ages 5 and 11.9 years the most [26], when parents have greater control, with no significant effect in adolescents. Adolescents should be empowered, by attending the SEP, to feel more confident in deciding their own insulin doses accurately and taking gradual care of their diabetes, under the supervision and support of their parents and the dietician, in preparation for transition later on into adult life [44]. Our findings show no significant effect of age and maternal education on the glycemic control at any time point beyond 6 months, which highlights the importance of continuous education beyond the initial phase after diagnosis [45].

The predictors of the optimal glycemic control at 12 months after diagnosis, including lower HbA1c at 6 months, fewer admissions due to hyperglycemia or DKA, higher maternal education and residence closer to our hospital, were significant in both the univariate and multivariate regression analyses. Tracking of HbA1c at 6 months to 12 months is expected [8], as shown in our results. More frequent admissions due to hyperglycemia or DKA, indicating lesser adherence, understandably predicted higher HbA1c at 12 months after diagnosis.

Higher maternal education being a significant predictor of lower HbA1c and lower incidence of acute and chronic complications [46] is obviously because their better knowledge and numeracy skills would enable more accurate calculation of carbohydrate content, insulin doses, and other aspects of diabetes care. Several studies had shown suboptimal glycemic control in PwD, who underestimated the carbohydrate content in their meals and hence underdosed insulin. For patients and/or caregivers with low levels of education or mathematical skills, the diabetes care team should try as much as possible to overcome these barriers, and modify diabetes education to enable them to calculate the carbohydrate content and insulin doses accurately under supervision [47]. Recent advances in diabetes-related technology, including online calculators and various applications, would be especially helpful for such families [48].

The closer place of residence predicting better glycemic control at 12 months after diagnosis was perhaps because this might ensure better supervision by the diabetes team [49]. However, there was no significant difference between the frequencies of clinic visits during the first year after diagnosis between the 2 groups.

We did not find gender a predictor of HbA1c at 12 months in the SEP group. Differences in HbA1c between genders, reported in the previous studies, might be because girls enter puberty earlier than boys, with consequent worsening of the glycemic control [50].

Age older than 12 years being a significant predictor in the univariate, but not in the multivariate analysis, indicates that other factors might affect glycemic control. Hawkes et al. did not find better HbA1c in adolescents after diabetes education programs [26].

Allocation of resources for structured programs and training of health care professionals, in developing countries, even in health care settings where dieticians are not available, could be cost-effective, with resultant better glycemic control and less devastating diabetes complications. Currently in Jordan, as in many other developing countries, there are no available validated structured diabetes education programs. Different regions in the country, and even hospitals in the same city, have variable clinical practices for delivering diabetes education to children with T1D and their families. The evidence our study provides of quality improvement in diabetes care in a developing country can be considered by health authorities and policy makers, who are implementing and supporting initiatives to improve diabetes outcomes in areas with limited resources. Individualized education and support should be delivered to PwD at high risk for suboptimal glycemic control. Our dietary education program needs modifications to adjust for high-risk patients, to achieve the intended goals and hopefully to be implemented nationally on a wider scale.

Strengths and Limitations. Our study is the first from Jordan to evaluate the effect of a structured dietary education program followed up over a period of 24 months after diagnosis. Further prospective research is needed to study the effect of programs such as ours on long-term glycemic control.

Our study had a number of limitations. First, it was a retrospective study, and hence the observed improvement in HbA1c levels might be not solely due to acquiring the skills of advanced carbohydrate counting but also other factors resulting in better adherence to the diabetes care plan, which we could not adjust for. Another limitation is that our study involved a single diabetes service in the capital Amman and did not represent the entire health services in Jordan. The PwD and caregivers who sought care at our specialized center might be more motivated and compliant to the diabetes care plan than others who were not. Wider studies involving differing health care settings and resource levels are needed. Our hospital is an academic center that owns relatively better resources and qualified specialized staff compared to other areas in the country. So, health care policies, in less-resourced settings, should aim to improve T1D care by training the health care professionals, who can provide PwD and caregivers the necessary skills.

## 5. Conclusion

Our structured dietary education program for newly diagnosed children and adolescents with T1D, and their caregivers, was associated with better glycemic control at 6 and 12 months after diagnosis, especially in PwD older than 5 years and with more educated mothers. Continuous diabetes education and close follow-up are needed to maintain the positive effects of the education program.

## Figures and Tables

**Table 1:** Demographic and socioeconomic characteristics during the first year after diagnosis of children in the structured education program (SEP) and those prior to the program (pre-SEP), *N* = 228.

	Pre-SEP *N* = 116 *N* (%)	SEP *N* = 112 *N* (%)	*P* value^*∗*^
Age at diagnosis			0.598
≤5 y	29 (25.0)	28 (25.0)	
5.1–11.9 y	68 (58.6)	60 (53.6)	
≥12 y	19 (16.4)	24 (21.4)	
Gender			0.866
Male	51 (44.0)	48 (42.9)	
Female	65 (56.0)	64 (57.1)	
Type of insurance			<0.001
Royal court	55 (47.4)	28 (25.0)	
Ministry of Health/University	61 (52.6)	84 (75.0)	
Family monthly income (Jordanian dinars)			0.547
<400	26 (22.4)	17 (15.2)	
400–800	74 (63.8)	76 (67.9)	
801–1200	8 (6.9)	10 (8.9)	
>1200	8 (6.9)	9 (8.0)	
Father education			0.878
Less than high school	13 (11.2)	9 (8.0)	
High school	32 (27.6)	34 (30.4)	
Higher than high school	69 (59.5)	68 (60.7)	
Dead	2 (1.7)	1 (0.9)	
Mother education			0.394
Less than high school	16 (13.8)	12 (10.7)	
High school	36 (31.0)	44 (39.3)	
Higher than high school	64 (55.2)	55 (49.1)	
Dead	0 (0)	1 (0.9)	
Living arrangements			0.373
With both parents	113 (97.4)	110 (98.2)	
With the father	0	1 (0.9)	
With the mother	3 (2.6)	1 (0.9)	
Place of residence			0.241
Capital city	76 (65.5)	87 (77.7)	
<70 km from capital city	26 (22.4)	16 (14.3)	
70–185 km from capital city	10 (8.6)	6 (5.4)	
>185 km from capital city	4 (3.4)	3 (2.7)	

^*∗*^Chi square test.

**Table 2:** Clinical characteristics during first year after diagnosis of children in the structured education program (SEP) and those prior to program start (pre-SEP), *N* = 228.

	Pre-SEP *N* = 116 *N* (%)	SEP *N* = 112 *N* (%)	*P* value^*∗*^
Who decides insulin dose?			0.006
Mother	108 (93.1)	101 (90.2)	
Father	5 (4.3)	0 (0.0)	
Patient herself/himself	2 (1.7)	11 (9.8)	
Sister/brother	1 (0.9)	0 (0.0)	
Diabetic ketoacidosis at diagnosis			0.424
Yes	60 (51.7)	52 (46.4)	
No	56 (48.3)	60 (53.6)	
Family history of 1^st^ degree relative with T1D			0.045
Yes	12 (10.3)	4 (3.6)	
No	104 (89.7)	108 (96.4)	
Number of blood glucose testing per day			<0.001
<4	31 (26.7)	0 (0.0)	
≥4	85 (73.3)	112 (100.0)	
Number of admissions due to diabetic ketoacidosis and hyperglycemia during 1^st^ year (excluding at diagnosis)			0.222
Zero	106 (91.4)	106 (94.6)	
1-2	7 (6)	6 (5.4)	
>2	3 (2.6)	0 (0.0)	
Number of admissions due to hypoglycemia during 1^st^ year			0.308
Zero	116 (100)	111 (99.1)	
1-2	0 (0)	1 (0.9)	
>2	0 (0)	0 (0)	
Number of admissions due to diabetic ketoacidosis and hyperglycemia during 2nd year			0.942
Zero	108 (93.1)	104 (92.9)	
1-2	8 (6.9)	8 (7.1)	
>2	0 (0)	0 (0)	
Number of admissions due to hypoglycemia during 2nd year			—
Zero	116 (100.0)	112 (100.0)	
1-2	0 (0)	0 (0)	
>2	0 (0)	0 (0)	
	Pre-SEP Mean ± SD	SEP Mean ± SD	*P* value^*∗∗*^
Body mass index standard deviation score at diagnosis	0.01 ± 1.35	0.02 ± 1.39	0.948
Number of clinic visits	4.59 ± 1.22	4.64 ± 1.77	0.779

^*∗*^Chi square test, ^*∗∗*^Independent samples *T* test.

**Table 3:** Association between HbA1c at different time intervals according to age groups and maternal educational level in structured education program (SEP) and pre-SEP.

	Pre-SEP		SEP		*P* value^*∗∗*^
	*N*	HbA1c (%) (mmol/mol)	*N*	HbA1c (%) (mmol/mol)	
All patients	116		112		
6 months		8.15 ± 1.56 (65.58 ± 17.05)		7.49 ± 1.19 (58.37 ± 13.00)	0.001
12 months		8.52 ± 1.82 (69.62 ± 19.90)		8.05 ± 1.46 (64.49 ± 15.95)	0.032
24 months		8.64 ± 1.55 (70.94 ± 16.94)		8.42 ± 1.58 (68.53 ± 17.27)	0.290
<5 y	29		28		
6 months		8.02 ± 1.26 (64.16 ± 13.77)		7.67 ± 1.33 (60.33 ± 14.54)	0.309
12 months		8.03 ± 1.51 (64.27 ± 16.50)		7.88 ± 1.27 (62.63 ± 13.88)	0.683
24 months		8.20 ± 1.37 (66.13 ± 14.97)		8.19 ± 1.78 (66.02 ± 19.45)	0.973
5.1–11.9 y	68		60		
6 months		8.03 ± 1.58 (64.27 ± 17.27)		7.45 ± 1.21 (57.93 ± 13.23)	0.021
12 months		8.54 ± 1.72 (69.84 ± 18.80)		8.07 ± 1.46 (64.71 ± 15.95)	0.095
24 months		8.73 ± 1.55 (71.92 ± 16.94)		8.43 ± 1.60 (68.64 ± 17.49)	0.286
≥12 y	19		24		
6 months		8.67 ± 1.85 (71.26 ± 20.22)		7.42 ± 0.94 (57.60 ± 10.27)	0.012
12 months		9.18 ± 2.42 (76.84 ± 26.45)		8.20 ± 1.68 (66.13 ± 18.36)	0.124
24 months		8.95 ± 1.75 (74.32 ± 19.13)		8.64 ± 1.27 (70.94 ± 13.87)	0.502
Less than high school	16		12		
6 months		8.57 ± 2.12 (70.17 ± 23.17)		8.08 ± 0.99 (64.81 ± 10.83)	0.419
12 months		9.52 ± 2.19 (80.55 ± 23.94)		8.73 ± 1.52 (71.92 ± 16.61)	0.294
24 months		9.35 ± 1.85 (78.70 ± 20.22)		9.27 ± 1.45 (77.82 ± 15.85)	0.903
High school	36		44		
6 months		8.24 ± 1.63 (66.56 ± 17.82)		7.73 ± 1.23 (60.99 ± 13.44)	0.119
12 months		8.61 ± 1.67 (70.61 ± 18.25)		8.12 ± 1.45 (65.25 ± 15.85)	0.165
24 months		8.80 ± 1.43 (72.68 ± 15.63)		8.61 ± 1.61 (70.61 ± 17.59)	0.586
Higher than high school	64		55		
6 months		7.97 ± 1.35 (63.61 ± 14.76)		7.17 ± 1.12 (54.87 ± 12.24)	0.001
12 months		8.22 ± 1.74 (66.34 ± 19.02)		7.84 ± 1.43 (62.19 ± 15.63)	0.207
24 months		8.36 ± 1.49 (67.87 ± 16.29)		8.06 ± 1.52 (64.60 ± 16.61)	0.276

^*∗∗*^Independent samples *T* test.

**Table 4:** Univariate and multivariate linear regression analyses of possible predictors of glycemic control (HbA1c %) at 12 months after diagnosis in the structured education program group.

	Univariate linear regression			Multivariate linear regression		
	*B*	95% CI	*P* value	*B*	95% CI	*P* value
Age at diagnosis						
≤5 y^*∗∗∗*^						
5.1–11.9 y	0.367	−0.154–0.887	0.167	0.354	−0.055–0.763	0.090
≥12 y	0.678	0.017–1.338	0.044	0.462	−0.131–1.056	0.126
Gender						
Male^*∗∗∗*^						
Female	−0.120	−0.5600.319	0.590	−0.018	−0.366–0.331	0.921
HbA1c at diagnosis	0.140	0.019–0.262	0.024	0.070	−0.030–0.170	0.167
HbA1c at 6 months	0.785	0.671–0.899	<0.001	0.700	0.576–0.823	<0.001
Family monthy income (Jordanian dinars)						
<400^*∗∗∗*^						
400–800	−0.740	−1.301–−0.179	0.010	−0.039	−0.508–0.431	0.871
801–1200	0.060	−0.850–0.970	0.896	0.222	−0.520–0.963	0.556
>1200	−0.663	−1.591–0.266	0.161	0.115	−0.634–0.863	0.763
Mother education						
Less than high school^*∗∗∗*^						
High school	−0.837	−1.546–−0.128	0.021	−0.499	−1.080–0.081	0.091
Higher than high school	−1.135	−1.813–−0.456	0.001	−0.568	−1.148–0.011	0.050
Dead	−1.378	−4.664–1.908	0.410	−1.183	−3.752–1.387	0.365
Place of residence						
Capital city^*∗∗∗*^						
<70 km from capital city	−0.153	−0.711–0.404	0.589	−0.135	−0.562–0.293	0.536
70–185 km from capital city	−0.249	−1.093–0.595	0.562	−0.005	−0.657–0.647	0.988
>185 km from capital city	2.090	0.846–3.333	0.001	0.955	−0.026–1.935	0.050
Who decides insulin dose mainly						
Mother^*∗∗∗*^						
Father	1.028	−0.459–2.514	0.174	−0.349	−1.579–0.881	0.577
Patient herself/himself	0.532	−0.407–1.471	0.266	0.121	−0.690–0.932	0.769
Sister/brother	0.770	−2.522–4.063	0.645	0.342	−2.191–2.875	0.790
Number of clinic visits 1^st^ year	0.039	−0.105–0.184	0.592	0.030	−0.082–0.142	0.601
Number of admission due to DKA and hyperglycemia during 1^st^ year						
Zero^*∗∗∗*^						
1-2	0.741	−0.170–1.652	0.110	0.572	−0.137–1.282	0.113
>2	3.502	1.649–5.356	<0.001	1.553	0.051–3.055	0.043

^*∗∗∗*^Reference; *B*: unstandardized coefficients.

## Data Availability

The data are available from the corresponding author upon on request.

## Supplementary Materials

Supplementary Materials Supplementary file 1: Dietary Education Program for Newly-Diagnosed Children with Type 1 Diabetes.

## References

[1] Brink S. J. (2001). Complications of pediatric and adolescent type 1 diabetes mellitus. *Current Diabetes Reports*.

[2] White N. H., Sun W., Cleary P. A. (2010). Effect of prior intensive therapy in type 1 diabetes on 10- year progression of retinopathy in the DCCT/EDIC: comparison of adults and adolescents. *Diabetes*.

[3] Danne T., Phillip M., Buckingham B. A. (2018). ISPAD clinical practice consensus guidelines 2018: insulin treatment in children and adolescents with diabetes. *Pediatric Diabetes*.

[4] Phelan H., Lange K., Cengiz E. (2018). ISPAD clinical practice consensus guidelines 2018: diabetes education in children and adolescents. *Pediatric Diabetes*.

[5] Ewers B., Vilsbøll T., Andersen H. U., Bruun J. M. (2019). The dietary education trial in carbohydrate counting (DIET-CARB Study): study protocol for a randomised, parallel, open-label, intervention study comparing different approaches to dietary self-management in patients with type 1 diabetes. *BMJ Open*.

[6] Kawamura T. (2007). The importance of carbohydrate counting in the treatment of children with diabetes. *Pediatric Diabetes*.

[7] Fu S., Li L., Deng S., Zan L., Liu Z. (2016). Effectiveness of advanced carbohydrate counting in type 1 diabetes mellitus: a systematic review and meta-analysis. *Scientific Reports*.

[8] Lawes T., Franklin V., Farmer G. (2014). HbA1c tracking and bio-psychosocial determinants of glycaemic control in children and adolescents with type 1 diabetes: retrospective cohort study and multilevel analysis. *Pediatric Diabetes*.

[9] DAFNE Study Group (2003). Training in flexible, intensive insulin management to enable dietary freedom in people with type 1 diabetes: dose adjustment for normal eating (DAFNE) randomized controlled trial. *Diabetic Medicine*.

[10] Bendik C. F., Keller U., Moriconi N. (2009). Training in flexible intensive insulin therapy improves quality of life, decreases the risk of hypoglycaemia and ameliorates poor metabolic control in patients with type 1 diabetes. *Diabetes Research and Clinical Practice*.

[11] Smart C. E., Annan F., Higgins L. A., Jelleryd E., Lopez M., Acerini C. L. (2018). Clinical practice consensus guidelines 2018: nutritional management in children and adolescents with diabetes. *Pediatric Diabetes*.

[12] Schmidt S., Schelde B., Nørgaard K. (2014). Effects of advanced carbohydrate counting in patients with type 1 diabetes: a systematic review. *Diabetic Medicine*.

[13] Chatterjee S., Davies M. J., Heller S., Speight J., Snoek F. J., Khunti K. (2018). Diabetes structured self-management education programmes: a narrative review and current innovations. *Lancet Diabetes & Endocrinology*.

[14] Ezenwaka C., Eckel J. (2011). Prevention of diabetes complications in developing countries: time to intensify self-management education. *Archives of Physiology and Biochemistry*.

[15] Jasper U. S., Opara M. C., Pyiki E. B., Akinrolie O. (2014). Knowledge of insulin use and its determinants among Nigerian insulin requiring diabetes patients. *Journal of Diabetes and Metabolic Disorders*.

[16] Caccavale L. J., Weaver P., Chen R., Streisand R., Holmes C. S. (2015). Family density and SES related to diabetes management and glycemic control in adolescents with type 1 diabetes. *Journal of Pediatric Psychology*.

[17] Steinke T. J., O’Callahan E. L., York J. L. (2017). Role of a registered dietitian in pediatric type 1 and type 2 diabetes. *Translational Pediatrics*.

[18] DiMeglio L., Acerini C., Codner E. (2018). ISPAD Clinical Practice Consensus Guidelines 2018: glycemic control targets and glucose monitoring for children, adolescents, and young adults with diabetes. *Pediatric Diabetes*.

[19] Grey M., Boland E. A., Davidson M., Li J., Tamborlane W. V. (2000). Coping skills training for youth with diabetes mellitus has long-lasting effects on metabolic control and quality of life. *Jornal de Pediatria*.

[20] Martin D., Lange K., Sima A. (2012). Recommendations for age-appropriate education of children and adolescents with diabetes and their parents in the European Union. *Pediatric Diabetes*.

[21] Giorgetti C., Ferrito L., Zallocco F., Iannilli A., Cherubini V., Study Group for Diabetes of ISPED (2015). Organization and regional distribution of centers for the management of children and adolescents with diabetes in Italy. *Italian Journal of Pediatrics*.

[22] Marden S., Thomas P. W., Sheppard Z. A., Knott J., Lueddeke J., Kerr D. (2012). Poor numeracy skills are associated with glycaemic control in Type 1 diabetes. *Diabetic Medicine*.

[23] Hayes R. L., Garnett S. P., Clarke S. L., Harkin N. M., Chan A. K., Ambler G. R. (2012). A flexible diet using an insulin to carbohydrate ratio for adolescents with type 1 diabetes—a pilot study. *Clinical Nutrition*.

[24] Gunn D., Mansell P. (2012 Jun). Glycaemic control and weight 7 years after dose adjustment for normal eating (DAFNE) structured education in type 1 diabetes. *Diabetic Medicine*.

[25] Mouslech Z., Somali M., Sarantis L. (2018). Significant effect of group education in patients with diabetes type 1. *Hormones*.

[26] Hawkes C. P., Willi S. M., Murphy K. M. (2019). A structured 1-year education program for children with newly diagnosed type 1 diabetes improves early glycemic control. *Pediatric Diabetes*.

[27] Essien O., Otu A., Umoh V., Enang O., Hicks J. P., Walley J. (2017). Intensive patient education improves glycaemic control in diabetes compared to conventional education: a randomised controlled trial in a Nigerian tertiary care hospital. *PLoS One*.

[28] Mash R. J., Rhode H., Zwarenstein M. (2014). Effectiveness of a group diabetes education programme in under-served communities in South Africa: a pragmatic cluster randomized controlled trial. *Diabetic Medicine*.

[29] Christie D., Thompson R., Sawtell M. (2014). Structured, intensive education maximising engagement, motivation and long-term change for children and young people with diabetes: a cluster randomised controlled trial with integral process and economic evaluation - the CASCADE study. *Health Technology Assessment*.

[30] Smart C. E., Ross K., Edge J. A., King B. R., McElduff P., Collins C. E. (2010). Can children with Type 1 diabetes and their caregivers estimate the carbohydrate content of meals and snacks?. *Diabetic Medicine*.

[31] Sullivan-Bolyai S., Bova C., Leung K., Trudeau A., Lee M., Gruppuso P. (2010). Social support to empower parents (STEP): an intervention for parents of young children newly diagnosed with type 1 diabetes. *Diabetes Educ. Jan-Feb*.

[32] Lindstrom C., Aman J., Norberg A. L. (2011). Parental burnout in relation to sociodemographic, psychosocial and personality factors as well as disease duration and glycaemic control in children with type 1 diabetes mellitus. *Acta Paediatrica*.

[33] Nwankwo C. U., Ezenwaka C. E., Onuoha P. C., Agbakoba N. R. (2015). Implementing diabetes self-management education (DSME) in a Nigerian population: perceptions of practice nurses and dieticians. *Archives of Physiology and Biochemistry*.

[34] Olamoyegun M. A., Iwuala S. O., Akinlade O. M., Olamoyegun K. D., Kolawole B. (2013). Assessment of diabetes related knowledge among health care providers in a tertiary health institution in Nigeria. *Nigerian Endocrine Practice*.

[35] Souto D. L., Zajdenverg L., Rodacki M., Rosado E. L. (2014). Impact of advanced and basic carbohydrate counting methods on metabolic control in patients with type 1 diabetes. *Nutrition*.

[36] Yeoh E., Choudhary P., Nwokolo M., Ayis S., Amiel S. A. (2015). Interventions that restore awareness of hypoglycemia in adults with type 1 diabetes: a systematic review and meta-analysis. *Diabetes Care*.

[37] Cryer P. E. (2002). Hypoglycaemia: the limiting factor in the glycaemic management of Type I and Type II diabetes. *Diabetologia*.

[38] Burghardt K., Müller U. A., Müller N. (2020). Adequate structured inpatient diabetes intervention in people with type 1 diabetes improves metabolic control and frequency of hypoglycaemia. *Experimental and Clinical Endocrinology & Diabetes*.

[39] Forsander G., Sundelin J., Persson B. (2007). Influence of the initial management regimen and family social situation on glycemic control and medical care in children with type I diabetes mellitus. *Acta Paediatrica*.

[40] Pendley J. S., Kasmen L. J., Miller D. L., Donze J., Swenson C., Reeves G. (2002). Peer and family support in children and adolescents with type 1 diabetes. *Journal of Pediatric Psychology*.

[41] Alassaf A., Gharaibeh L., Odeh R., Ibrahim S., Ajlouni K. (2022). Predictors of glycemic control in children and adolescents with type 1 diabetes at 12 months after diagnosis. *Pediatric Diabetes*.

[42] Haller M. J., Satalvey M. S., Silverstein J. H. (2004). Predictors of control of diabetes: monitoring may be the key. *Jornal de Pediatria*.

[43] D’Souza R. S., Ryan M., Hawkes E. (2021). Questionnaire-based service evaluation of the efficacy and usefulness of SEREN: a structured education programme for children and young people diagnosed with type 1 diabetes mellitus. *BMJ Open Qual*.

[44] Hsin O., La Greca A. M., Valenzuela J., Moine C. T., Delamater A. (2010). Adherence and glycemic control among Hispanic youth with type 1 diabetes: role of family involvement and acculturation. *Journal of Pediatric Psychology*.

[45] Cabrera S. M., Srivastava N. T., Behzadi J. M., Pottorff T. M., Dimeglio L. A., Walvoord E. C. (2013). Long-term glycemic control as a result of initial education for children with new onset type 1 diabetes: does the setting matter?. *The Diabetes Educator*.

[46] Al-Odayani A. N., Alsharqi O. Z., Ahmad A. M., Khalaf Ahmad A. M., Al-Borie H. M., Qattan A. M. (2013). Children’s glycemic control: mother’s knowledge and socioeconomic status. *Global Journal of Health Science*.

[47] Kawamura T., Takamura C., Hirose M. (2015). The factors affecting on estimation of carbohydrate content of meals in carbohydrate counting. *Clinical Pediatric Endocrinology*.

[48] Huckvale K., Adomaviciute S., Prieto J. T., Khee-Shing Leow M., Car J. (2015). Smartphone apps for calculating insulin dose: a systematic assessment. *BMC Medicine*.

[49] Zuijdwijk C. S., Cuerden M., Mahmud F. H. (2013). Social determinants of health on glycemic control in pediatric type 1 diabetes. *Jornal de Pediatria*.

[50] Samuelsson U., Anderzen J., Gudbjornsdottir S., Steineck I., Akesson K., Hanberger L. (2016). Teenage girls with type 1 diabetes have poorer metabolic control than boys and face more complications in early adulthood. *Journal of Diabetic Complications*.

