# Supplementary material for: Effect on Glycemic Control of an Early Intensive Dietary Structured Education Program for Newly Diagnosed Children with Type 1 Diabetes in Jordan
Source: Pediatr Diabetes. 2023 Apr 28;2023:7258136. doi: 10.1155/2023/7258136 (PMC12017027; doi:10.1155/2023/7258136)
Supplement: Supplementary Materials — Supplementary file 1: Dietary Education Program for Newly-Diagnosed Children with Type 1 Diabetes. [file 7258136.f1.pdf]

Dietary Education Program for Newly-Diagnosed Children with Type 1 Diabetes

Pediatric Endocrinology Division/ Jordan University Hospital

For newly-diagnosed children and adolescents with type 1 diabetes (T1D), a nutrition assessment for the weight, height and the dietary habits is done by the pediatric dietician, who then demonstrates to the parents and caregivers how the dietary regimen affects blood glucose levels. In addition to illustrating the energy sources in food and the macronutrients (carbohydrate, protein and fat), the main carbohydrate sources and its significant effect on blood glucose levels; bearing in mind the other factors that may affect the blood glucose level, as physical activity and sickness. During the stay at hospital, we provide an intensive education program to give the parents sufficient knowledge to deal with their child according to his/her dietary habits, life style, and physical activity.

Our education program starts gradually from the day of admission to the day of discharge by:

1. Nutritional assessment.
2. Calculating the total daily energy requirements.
3. Demonstrating the carbohydrate sources and types in food and explain the differences between low and high glycemic index and how each type has different effect on blood glucose level.
4. Discussing the method of carbohydrate counting with the patient and parents, taking into consideration the parents' educational level, by using the scale to weigh the food items or using portions, and determination of the carbohydrate amount using carbohydrate food list.
5. On day one or two, we start to teach the parents how to count carbohydrates for every meal, and how to calculate the insulin doses accordingly. On day two or three the

parents start to count carbohydrate and calculate the insulin dose on their own under the supervision of the dietitian.

6. Demonstrating to parents how to deal with deranged blood glucose levels (hypoglycemia and hyperglycemia).
7. Demonstrating how to adjust insulin doses according to the trends in blood glucose levels.

Any questions or concerns from patients and parents are discussed during the hospital stay, to make sure they will be able to deal with diabetes properly.

During the first week after discharge, there will be mutual contact between the dietitian and the parents for follow up of blood glucose readings and to ensure that the concept of carbohydrate count is delivered properly.

The patient is scheduled for pediatric endocrine clinic visit one week after discharge, and the dietitian clinic for check up and reviewing the blood glucose level readings, and further clinic visits are scheduled at two, four and 8 weeks after the hospital discharge.

Afterwards, regular clinic visits to be scheduled every 3 months, and then when needed in between those visits. Contact with dietician after the third month, becomes as needed for any question regarding insulin dose calculation and adjustment. Beyond the first year after diagnosis, patients are continued to be seen at the pediatric endocrinology clinic every 3 months and to be seen by the dietician at least twice per year; to reinforce knowledge and skills.
